# Next-generation sequencing yields the complete chloroplast genome of *C. goeringii* acc. smg222 and phylogenetic analysis

**DOI:** 10.1080/23802359.2018.1437812

**Published:** 2018-02-12

**Authors:** Heng Wang, So-Yeon Park, Ah-Rim Lee, Seong-Gyu Jang, Da-Eun Im, Tae-Hwan Jun, Joohyun Lee, Jong-Wook Chung, Tae-Ho Ham, Soon-Wook Kwon

**Affiliations:** a Department of Plant Bioscience, College of Natural Resources and Life Science, Pusan National University, Miryang, Republic of Korea;; b Department of Applied Bioscience, Konkuk University, Seoul, Republic of Korea;; c Department of Industrial Plant Science and Technology, Chungbuk National University, Cheongju, Republic of Korea;; d Department of Agricultural Science, Korea National Open University, Seoul, Republic of Korea

**Keywords:** *Cymbidium goeringii* acc. smg222, chloroplast genome, Illumina sequencing

## Abstract

Spring orchid (*Cymbidium goeringii*) is one of the most important species belonging to *Orchidaceae* owing to its aesthetic appeal, fragrant flowers and ideal characteristics for using as a houseplant. In this study, the complete chloroplast genome of Korean *C. goeringii* acc. smg222 was determined by Illumina sequencing. The circular double-stranded DNA of 148,441 bp consisted of two inverted repeat regions of 25,610 bp each, a large single copy region of 83,311 bp, and a small single copy region of 13,910 bp. The genome contained 122 genes, of which 104 were unique and 18 were duplicated within the IRs. The 104 unique genes included 70 protein-coding genes, 30 distinct tRNA genes, and four rRNA genes. Phylogenetic tree analysis revealed that *C. goeringii* acc. smg222 was clustered with *Cymbidium kanran*, a cymbidium species native to Korea.

*Cymbidium* orchids (*Orchidaceae*) are among the most popular orchids worldwide and it is distributed in tropical, subtropical Asia and northeastern Australia (Yang et al. 2013). Spring orchid (*C. goeringii*) is one of the most important species belonging to *Cymbidium* (Wang et al. 2009) owing to its aesthetic appeal and fragrant flowers for using as a decorative pot flowers (Liu et al. 2014). During the long cultivation history, most of the commercially important and attractive cymbidiums have been produced by selection on somatic mutations during vegetative propagation and hybrids derived from other species (Wang et al. 2009; Liu et al. 2014). These currently recognized variants are often complex, involving several species in their ancestry (Obara-Okeyo and Kako 1998). Chloroplasts, as one of the essential organelles, have become an effective method for phylogenetic analysis (Parks et al. 2009). Here, we report the complete chloroplast genome of *C. goeringii* acc. smg222, a spring orchid native to Korea, and generated phylogenetic tree to provide an insight into genetic relationships of *Cymbidium* and related species.

Total genomic DNAs were extracted from mature leaves obtained from the Saemangeum Bio Center, Republic of Korea (35°56′31.2″N, 126°48′53.0″E). An Illumina paired-end DNA library (average insert size of 500 bp) was constructed and sequenced by Illumina HiSeq platform. Totally, 2.2 Gb paired-end reads were obtained and assembled using the CLC Genome Assembler (version beta 4.6; CLC Bio, Aarhus, Denmark) with a minimum overlap size of 200 bp and maximum bubble size of 50 bp for the de Bruijn graph. Chloroplast contigs were selected from the initial assembly by performing a BLAST (version 2.2.31) search against the reference chloroplast genome of *C. kanran* (GenBank accession: KU179435) using CLC. The complete chloroplast genome was annotated and assembled with Dual Organellar GenoMe Annotator (DOGMA) (Wyman et al. 2004) and CpGAVAS (Liu et al. 2012). The whole chloroplast genome sequence has been deposited to NCBI GenBank with the accession number of MF421552.

The *C. goeringii* acc. smg222 chloroplast genome was composed of a single, circular double-stranded DNA molecule, consisting of a pair of IRs (25,610 bp) separated by the LSC (83,311 bp) and SSC (13,910 bp) regions. The overall GC content was 37.1%. The chloroplast genome contains 122 genes, of which 104 were unique and 18 were duplicated in the IR regions. The 104 unique genes included 70 protein-coding, 30 transfer RNA, and four ribosomal RNA genes.

We also constructed a phylogenetic analysis with the complete chloroplast genome of *C. goeringii* acc. smg222 and 11 other species from the family of *Cymbidium* (Figure 1). The neighbour-joining phylogenetic tree analysis was performed using MEGA 7.0 (Kumar et al. 2016). The tree showed that *C. goeringii* acc. smg222 was subgrouped with *C. kanran*, a Korean landrace (Chung et al. 1985). The newly characterized *C. goeringii* acc. smg222 chloroplast genome reported here provides a useful tool not only for studying of the evolutionary history of *Cymbidium*, but also for population structure and phytogeographic studies for *Cymbidium*.

**Figure 1.**
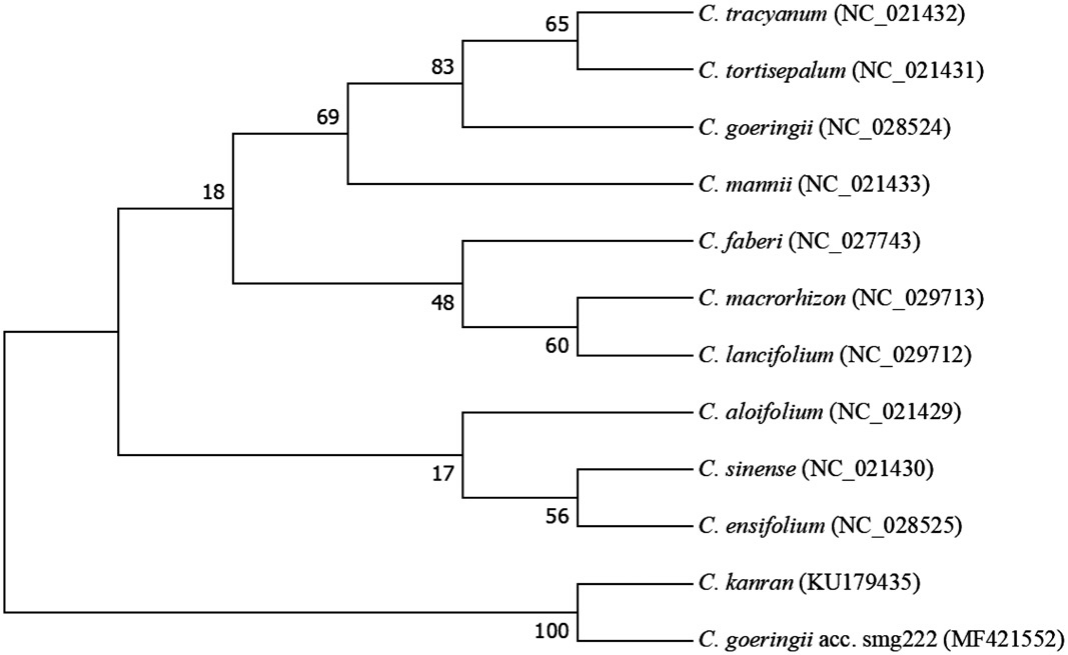
Neighbour-joining phylogenetic tree based on 12 complete chloroplast genome sequences of *Cymbidium* family. Numbers in the nodes indicate the bootstrap support values from 2000 replicates.
